# Beta-Caryophyllene Modifies Intracellular Lipid Composition in a Cell Model of Hepatic Steatosis by Acting through CB2 and PPAR Receptors

**DOI:** 10.3390/ijms24076060

**Published:** 2023-03-23

**Authors:** Rosaria Scandiffio, Sara Bonzano, Erika Cottone, Sujata Shrestha, Simone Bossi, Silvia De Marchis, Massimo E. Maffei, Patrizia Bovolin

**Affiliations:** 1 Cell Biology Unit, Department of Life Sciences and Systems Biology, University of Turin, Via Accademia Albertina 13, 10123 Turin, Italy; 2 Plant Physiology Unit, Department of Life Sciences and Systems Biology, University of Turin, Via Quarello 15/a, 10135 Turin, Italy; 3 Neuroscience Institute Cavalieri Ottolenghi (NICO), Regione Gonzole 10, Orbassano, 10043 Turin, Italy

**Keywords:** β-caryophyllene, NAFLD, steatosis, lipid profile, CB2 receptors, PPARγ, PPARα, HepG2 cell line

## Abstract

Non-alcoholic fatty liver disease (NAFLD) is the most common cause of chronic liver disease; however, no specific pharmacological therapy has yet been approved for this condition. Plant-derived extracts can be an important source for the development of new drugs. The aim of this study was to investigate the effects of (E)-β-caryophyllene (BCP), a phytocannabinoid recently found to be beneficial against metabolic diseases, on HepG2 steatotic hepatocytes. Using a fluorescence-based lipid quantification assay and GC-MS analysis, we show that BCP is able to decrease lipid accumulation in steatotic conditions and to change the typical steatotic lipid profile by primarily reducing saturated fatty acids. By employing specific antagonists, we demonstrate that BCP action is mediated by multiple receptors: CB2 cannabinoid receptor, peroxisome proliferator-activated receptor α (PPARα) and γ (PPARγ). Interestingly, BCP was able to counteract the increase in CB2 and the reduction in PPARα receptor expression observed in steatotic conditions. Moreover, through immunofluorescence and confocal microscopy, we demonstrate that CB2 receptors are mainly intracellularly localized and that BCP is internalized in HepG2 cells with a maximum peak at 2 h, suggesting a direct interaction with intracellular receptors. The results obtained with BCP in normal and steatotic hepatocytes encourage future applications in the treatment of NAFLD.

## 1. Introduction

Non-alcoholic fatty liver disease (NAFLD) is the most common chronic liver disorder, with an average prevalence of 25%, ranging from 13% (adult African population) to 32% (Middle East population) [1]. This disease is characterized by the excessive accumulation of fats, due to overnutrition or an unbalanced diet and not to ethanol consumption, with an increase of visceral fats that results in macrophage infiltration and pro-inflammatory conditions [2]. In this context, insulin resistance occurs, causing dysregulated lipolysis of triglyceride in the adipose tissue and delivery of fats to the liver. This is accompanied by increased de novo lipogenesis, the process through which hepatocytes convert excess carbohydrates to free fatty acids (FFA). The disposal of FFA occurs through beta-oxidation or re-esterification, and when this process is overloaded the formation of lipotoxic lipids may occur. This causes oxidative stress, endoplasmic reticulum stress and hepatocellular damages. The exacerbation of this condition leads to nonalcoholic steatohepatitis (NASH) that can progress to cirrhosis and liver cancer [1,2]. No specific pharmacological treatments are currently approved for NAFLD and NASH. The therapeutic strategies are based on lifestyle improvement (i.e., physical activity and healthy diet) associated with periodical checking of cardiometabolic risk factors to avoid advanced forms of NAFLD and the prevention of complications [3]. Nevertheless, numerous anti-hypertensive, lipid-lowering (statins) and glucose-lowering drugs (metformin) have been investigated because of NAFLD association with type 2 diabetes (T2DM), hypertension, obesity and dyslipidemia [3]. Other investigated drugs are those belonging to peroxisome proliferator-activated receptor (PPAR)γ agonists (e.g., pioglitazone, rosiglitazone). However, because of the side effects, the incomplete efficacy of these drugs and the variability of the conditions that exist among different patients, the suggested strategies remain those mentioned above [3].

In recent years, natural molecules have been demonstrated to ameliorate typical conditions of NAFLD, from steatosis to inflammation. Among them, the methyl brevifolincarboxylate, a polyphenolic compound, can reduce inflammation and oxidative stress in an hepatocarcinoma cell line [4] and berberine, a benzylisoquinoline alkaloid, can reduce triglyceride synthesis-related genes in in vitro and in vivo models [5]; nevertheless these molecules have not yet been approved by the FDA. Instead, (E)-β-caryophyllene (BCP) has been recognized by the FDA as a safe food or cosmetic additive. BCP is a bicyclic sesquiterpene hydrocarbon widely distributed in the plant kingdom, especially in floral volatiles, occurring in more than 50% of angiosperm families [6]. In plants, BCP acts as a chemoattractant for pollinators, defense against bacterial pathogens and has a pivotal role in the survival and evolution of higher plants as well as in contributing to the unique aroma of essential oils extracted from numerous species [7]. In addition to its role in plants, recent studies have highlighted that BCP plays a role in animal cells as anti-cancer [8], anti-oxidant [9], anti-inflammatory agent [10]. Although its mechanism of action is not yet fully understood, studies indicate that BCP could act in animal cells through the specific binding to the CB2 cannabinoid receptors [11,12], of which it is a full selective agonist, and possibly through the interaction with members of the family of peroxisome proliferator-activated receptor (PPAR), in particular the isoforms α and γ [13,14].

CB2 receptors belong to the endocannabinoid system (ECS), a complex endogenous system involved in several physiological and pathophysiological functions. The ECS exerts regulatory control on metabolism and food intake and for this reason it represents a potential target for numerous metabolic disorders such as obesity, eating disorders, dyslipidemia and steatosis [15,16]. The ECS is also involved in the regulation of inflammation and in the modulation of depression, schizophrenia and chronic pain [17,18]. The selectivity of BCP for CB2 receptors avoids potential psychotropic effects mediated by the neuronal CB1 cannabinoid receptor, being CB2 receptors mainly expressed in peripheral tissues and in central nervous system (CNS) immune cells [19]. This peculiarity makes BCP a safe phytocannabinoid, with countless beneficial and non-psychoactive effects.

PPAR nuclear receptors are transcriptional modulators, with each isoform having a specific location and role regarding energy homeostasis, lipid and glucose metabolism and inflammatory response [20]. PPARα is mainly expressed in the liver (but also in brown adipose tissue, heart, muscles and kidney) and acts as the master regulator of hepatic lipid metabolism, being involved especially in fatty acid (FA) beta-oxidation. PPARγ is characterized by three isoforms: PPARγ1, PPARγ2 and PPARγ3. PPARγ1 is ubiquitously expressed, PPARγ2 is mainly expressed in adipose tissue and in the liver, whereas PPARγ3 is expressed in the colon and adipose tissue [20]. The role of the three isoforms slightly changes based on cell type, but in the liver they are essentially involved in the regulation of glucose and lipid metabolism, protection against inflammation, oxidation and liver fibrosis [21].

The interaction between BCP and PPARα and PPARγ is not as well documented as it is for CB2 receptors; however, some studies have shown that there is a direct interaction between BCP and PPARα [13] and probably an indirect interaction with PPARγ. In fact, the triggering of PPARγ via BCP-mediated CB2 receptor activation has been hypothesized [22].

This study aimed to investigate the anti-steatotic effects of BCP in the immortalized human hepatoma cell line HepG2, one of the cell models mostly used to induce NAFLD and to test the therapeutic effects of pharmacological and natural compounds [23]. To mimic the key NAFLD risk factor, increased fat intake, we added palmitic and oleic acid to cell culture media obtaining significant intracellular lipid accumulation in the absence of overt cytotoxicity. We first investigated the effect of BCP on the intracellular lipid accumulation and lipid profile, then we examined the involvement of different receptors in these processes. Our findings suggest that treatment with BCP induces a reduction in lipid accumulation and a modification in intracellular lipid composition mediated by CB2 and PPAR receptors and that these effects are accompanied by a modulation of their expression. We also observed that BCP is able to cross the plasma membrane and therefore to act on intracellular localized receptors.

## 2. Results

### 2.1. HepG2 Cell Viability Is Not Affected by Steatosis Induction and BCP Treatment

HepG2 hepatoma cells were induced to become steatotic by 24 h treatment with 0.5 mM FFA mixture, made of sodium palmitate and sodium oleate (1:2, *w*/*w*, referred hereafter as FFAm) along with BSA (1%, *w*/*v*).

**Figure 1:**
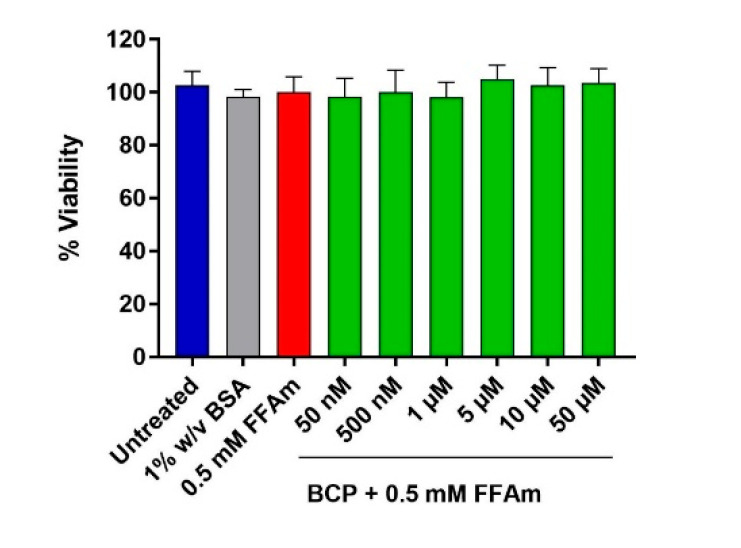
Cell viability assay of HepG2 cells based on ATP content. HepG2 cells were treated for 24 h with 0.5 mM FFAm and with increasing concentrations of BCP. Either BCP plus 0.5 mM FFAm, FFAm alone or 1% *w*/*v* BSA did not affect HepG2 cell viability. Control conditions (untreated) and treatments are represented as the mean ± SEM of three independent experiments.

To analyze BCP effect on cell viability, HepG2 cells were treated for 24 h with 0.5 mM FFAm alone or in the presence of different concentrations of BCP (50 nM, 500 nM, 1 µM, 5 µM, 10 µM and 50 µM). Untreated cells and cells treated with 1% BSA only were used as controls. The CellTiter-Glo^®^ viability assay shows that neither FFAm nor FFAm + BCP affect HepG2 steatotic cell viability (Figure 1). 

### 2.2. BCP Reduces Intracellular Triglyceride Content in HepG2 Steatotic Cells 

**Figure 2:**
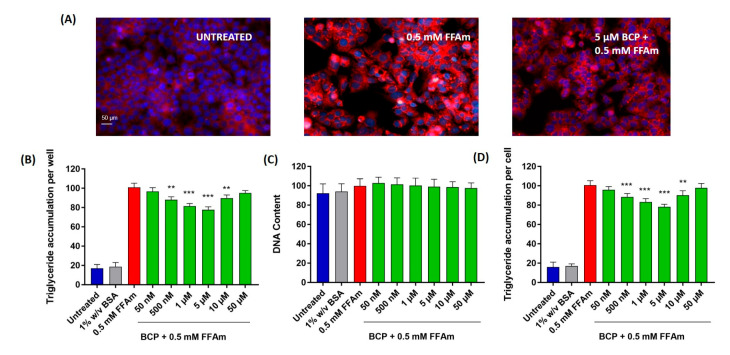
Lipid accumulation in HepG2 cells. BCP attenuates intracellular lipid accumulation in steatotic HepG2 cells without altering the cell number (DNA content). (**A**) Representative images of AdipoRed (red, triglycerides) and NucBlue (blue, nuclei) stainings; UNTREATED = untreated hepatocytes (control); 0.5 mM FFAm = palmitate and oleate-treated hepatocytes; 5 μM BCP + 0.5 mM FFAm = HepG2 cells co-treated for 24 h with BCP and FFAm. Scale bar: 50 μm. (**B**) Bar graph summarizing AdipoRed staining experiments to assess triglyceride accumulation per well in untreated cells, BSA-treated cells, 0.5 mM FFAm-treated positive control cells and HepG2 cells treated with 0.5 mM FFAm and various concentrations of BCP for 24 h. (**C**) Bar graph summarizing the DNA content per well (NucBlue staining). (**D**) Bar graph showing the triglyceride accumulation per cell, calculated as the ratio of AdipoRed and NucBlue stainings. Data are expressed as percentage change with respect to 0.5 mM FFA control condition (set equal to 100) and represent the mean ± SEM of five independent experiments. ** *p* < 0.01; *** *p* < 0.001 vs. positive control (0.5 mM FFAm-treated cells).

To study the effect of BCP on the induction of steatosis, HepG2 cells were co-treated with 0.5 mM FFAm and increasing concentrations of BCP (ranging from 50 nM to 50 µM). After 24 h treatment, lipid droplets and nuclei were visualized by AdipoRed^TM^/NucBlue^TM^ fluorescent staining (Figure 2A). Triglyceride accumulation and DNA content were quantified and expressed as a percentage change with respect to 0.5 mM FFAm-treated cells (positive control) (Figure 2B–D). 

Triglyceride accumulation was highly increased by the treatment with FFAm, compared to untreated control cells, while the co-treatment with 500 nM, 1 µM, 5 µM and 10 µM BCP significantly (*p* < 0.01) reduced the amount of intracellular triglycerides (Figure 2B). This result was not due to cytotoxic effects or to a reduction in HepG2 cell proliferation, since the treatment with FFAm and BCP did not induce any significant change in the DNA content (Figure 2C), nor in the cell viability (Figure 1), in respect to untreated and FFAm-treated cells. No changes in triglyceride accumulation or the DNA content were observed in cells cultured in the presence of 1% *w*/*v* BSA alone. 

The DNA content was used to normalize the total triglyceride values to obtain the triglyceride content per unit DNA (as a proxy for triglycerides accumulation per cell). Figure 2D shows that 500 nM, 1 µM, 5 µM and 10 µM BCP were able to induce a significant (*p* < 0.01) decrease in triglyceride accumulation/cell, with the maximum reduction at 5 µM (corresponding to a 22% reduction of the levels of the FFAm positive control). 

### 2.3. BCP Modifies the Intracellular Lipid Profile of HepG2 Steatotic Cells 

**Figure 3:**
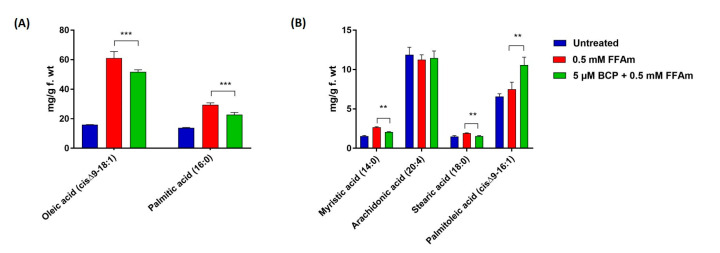
FFA composition of HepG2 cells after incubation for 24 h with 0.5 mM FFAm with or without BCP. Six fatty acids were identified and quantified. (**A**) BCP reduces the amount of oleic acid (cis-Δ9-C18:1) and palmitic acid (C:16) of steatotic cells. (**B**) BCP treatment significantly reduces the content of myristic acid (C14:0) and stearic acid (C18:0), while it increases palmitoleic acid (cis-Δ9-C16:1), in comparison to steatotic control cells. Data are represented as the mean ± SEM of three independent experiments and the values are expressed as mg g−1 fresh weight (f.wt). ** *p* < 0.01; *** *p* < 0.001 vs. positive control (0.5 mM FFAm-treated cells).

To evaluate whether the effect of BCP on total lipid content could be linked to changes in the intracellular lipid profile, GC-MS was employed to identify specific intracellular FFA, whereas GC-FID was used for the quantitative analysis. The FFA composition of steatotic HepG2 cells (0.5 mM FFAm-treated cells) was consistent with that of typical NAFLD models, including myristic acid (C14:0), palmitic acid (C16:0), palmitoleic acid (*cis-*Δ^9^-C16:1), stearic acid (C18:0), oleic acid (*cis*Δ^9^-C18:1) and arachidonic acid (C20:4). Compared to untreated controls, steatotic HepG2 cells (Figure 3, red bars) showed a statistically significant (*p* < 0.01) increase of *cis-*Δ^9^-C18:1 and C16:0 (Figure 3A), as well as C14:0, C18:0 and *cis-*Δ^9^-C16:1 (Figure 3B); no significant (*p* > 0.05) changes were found for C20:4 (Figure 3B). Treatment of steatotic cells with BCP (Figure 3, green bars) caused a significant (*p* < 0.01) reduction (−22%) in the amount of *cis*-Δ^9^-C18:1 and C16:0 (Figure 3A), compared to 0.5 mM FFAm-treated cells; the same reduction was found for C14:0, whereas a 19% reduction occurred for C18:0 (Figure 3B). Interestingly, a significant 37% increase was found for *cis*-Δ^9^-C16:1. No changes were found for C20:4 (*p* > 0.05) (Figure 3B). These results indicate that BCP was able to reduce the amount of all the identified saturated FFA, while the unsaturated C20:4 was unaffected and the levels of the unsaturated *cis*-Δ^9^-C16:1 were increased by BCP treatment. 

### 2.4. BCP Inhibits Lipid Accumulation through Interaction with Different Receptors: Effects of CB2 and PPAR Receptor Antagonists

**Figure 4:**
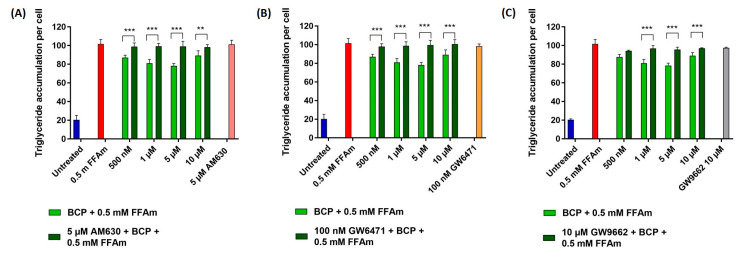
Effect of CB2, PPARα and PPARγ receptors antagonists on triglyceride accumulation per cell. Cells were incubated with different concentrations of BCP and 0.5 mM FFAm, in the presence or absence of specific receptor antagonists for 24 h. (**A**) Treatment of HepG2 cells with 5 µM CB2 antagonist AM630. (**B**) Treatment with 100 nM PPARα antagonist GW6471. (**C**) Treatment with 10 µM PPARγ antagonist GW9662. Data are expressed as a percentage change with respect to 0.5 mM FFA control condition (set equal to 100) and represent the mean ± SEM of five independent experiments. ** *p* < 0.01; *** *p* < 0.001 vs. BCP + 0.5 mM FFAm treated cells.

In order to characterize the mechanism of action of BCP in the reduction of lipid accumulation in steatotic HepG2 cells, a receptor antagonist approach was employed. In particular, we focused on cannabinoid CB2 receptors and receptors involved in lipid metabolism, i.e., PPARα and PPARγ. The specific CB2 receptor antagonist AM630, the PPARα receptor antagonist GW6471 and the PPARγ antagonist GW9662 were used at concentrations obtained from literature data [11,24,25]. No effects on lipid accumulation (Figure 4A–C) nor on cell viability were observed when treating HepG2 cells with each of the different antagonists alone, AM630, GW6471 and GW9662.

At the concentration range between 500 nM and 10 µM BCP (identified as the most effective doses in previous experiments), the co-treatment of HepG2 cells with the CB2 receptor antagonist AM630 (5 µM) completely reversed BCP-driven reduction of lipid accumulation, restoring values of intracellular triglycerides comparable to that of steatotic cells (Figure 4A). Similarly, treatment with the PPARα antagonist GW6471 (100 nM) completely reversed the anti-steatotic effect of BCP at all concentrations (Figure 4B). The treatment with the PPARγ-specific antagonist GW9662 (10 µM) partially reversed the lipid reduction induced by 1, 5 and 10 µM BCP (Figure 4C).

These results indicate that BCP is able to reduce lipid accumulation in steatotic HepG2 cells by interacting with CB2 and PPAR receptors.

### 2.5. CB2, PPARα and PPARγ mRNA Expression Is Affected by Steatosis and BCP Treatment 

**Figure 5:**
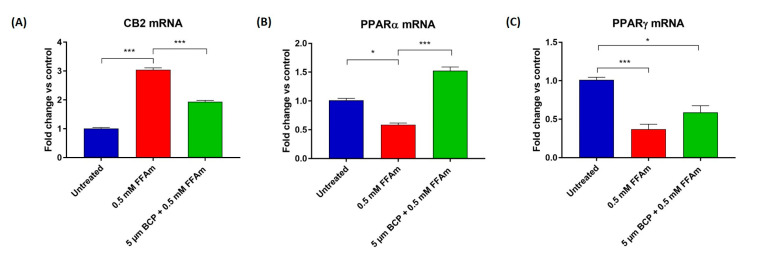
qRT-PCR analysis of CB2 (**A**), PPARα (**B**) and PPARγ (**C**) mRNAs normalized for the housekeeping gene β-actin. Data are represented as the mean ± SEM of three independent experiments. * *p* < 0.05, *** *p* < 0.001 vs. control (untreated cells, set equal to 1) or vs. 0.5 mM FFAm-treated cells.

To investigate whether CB2, PPARα and PPARγ mRNA expression levels are modified by steatosis and the ability of BCP to revert these changes, qRT-PCR experiments were conducted. The expression level of the *CNR2* gene resulted in significantly upregulated steatotic cells, when compared to untreated cells. Interestingly, co-treatment with BCP significantly reduced (*p* < 0.001) CB2 mRNA levels in steatotic cells, bringing CB2 expression levels closer to those of the non-pathological condition (Figure 5A). 

The expression of PPARs was significantly reduced in steatotic conditions compared to untreated cells (Figure 5B,C). Co-treatment of steatotic cells with BCP resulted in a significant (*p* < 0.001) increase in PPARα expression (Figure 5B), while no statistically significant change was observed in the expression of PPARγ (Figure 5C).

### 2.6. CB2 Receptors Are Localized Intracellularly in HepG2 Cells 

**Figure 6:**
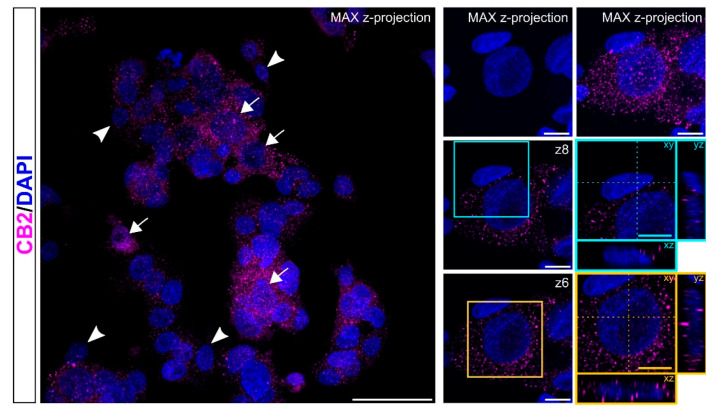
Localization of CB2 receptors. Representative confocal images showing CB2 immunostaining (magenta) in HepG2 cell line. Nuclei are stained with DAPI (blue). Pictures are shown as max z-projections (low magnification; **left**) with white arrows and arrowheads to highlight CB2high+ cells and CB2low+ cells, respectively, and a single confocal plane with reslicing (**right**) to better appreciate the intracellular distribution of CB2+ puncta in two of the cells present in the image. The cyan contoured image shows a cell positive for CB2 at low levels; the yellow contoured image identifies a cell with extensive immunolabelling for CB2. Scale bars: 50 µm (low magnification) and 10 µm (high magnification).

Since data on CB2 receptor localization are lacking in hepatocytes, we performed CB2 immunofluorescence experiments on HepG2 cells. Figure 6 shows a confocal image with punctate staining mainly located at intracellular sites. The staining level for CB2 in HepG2 cells appears quite heterogeneous, as evidenced by the presence of CB2high+ cells (arrows) and CB2low + (arrowheads) cells in the same clusters of cells (Figure 6, left panel). Single confocal planes show a prevalent perinuclear distribution of CB2 immunopositive puncta (Figure 6, right panels).

### 2.7. BCP Enters HepG2 Cells with a Maximum Uptake at 2 h from the Beginning of Treatment

**Figure 7:**
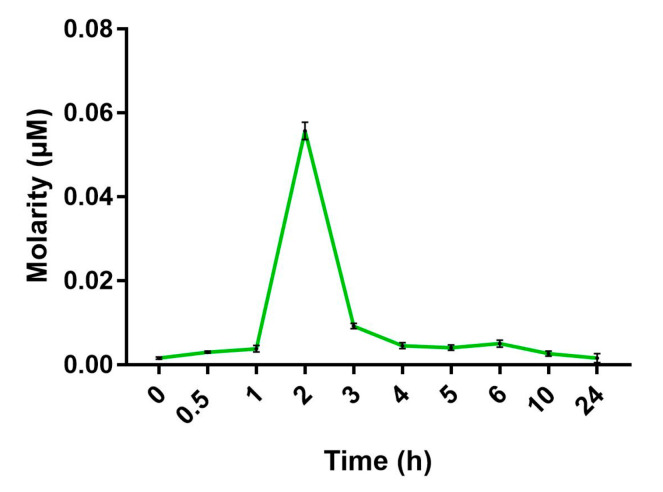
Time-course of BCP uptake by HepG2 cells measured with GC-MS. Maximum concentration of BCP was found 2 h after the beginning of the treatment. Data are represented as the mean ± SEM of three independent experiments.

Since we demonstrated that CB2 receptors are mostly intracellularly localized and PPAR receptors are well-known nuclear receptors, we decided to assess whether BCP is indeed able to enter HepG2 cells. The quantification of BCP intracellular uptake was evaluated by a time-course analysis in living cells by GC-MS. HepG2 cells were treated with 5 µM BCP for 24 h and samples were taken from time 0 to 24 h after treatment. By using GC-MS in the single ion monitoring (SIM) for BCP ions, we found that BCP was able to cross the cell membrane and enter HepG2 cells as soon as 1 h from the beginning of the treatment, with a maximum uptake measured at 2 h. After this period, the BCP intracellular concentration decreased linearly up to 24 h after treatment (Figure 7). 

## 3. Discussion

Despite recent progresses in understanding the various steps involved in the development and progression of NAFLD, no approved pharmacological treatments for this very common chronic disease are yet available [3]. Therefore, additional efforts are needed to find molecules able to interact with the molecular targets identified in NAFLD pathogenesis. Plant-derived molecules can be an important source for the development of new drugs [26]; in this work, we focused our attention on the anti-steatotic activity of BCP, a phytocannabinoid with promising therapeutic effects in metabolic disorders and inflammation. 

In order to mimic NAFLD in vitro, we incubated HepG2 hepatocytes for 24 h with a mixture of oleate and palmitate, which are the most abundant monounsaturated and saturated FFA in human diet [23]. Our steatosis protocol differed from the one used by Kamikubo and colleagues [24], who incubated HepG2 cells with palmitic acid only, a treatment that generally induces higher toxicity and the release of pro-inflammatory chemokines, which are typical of NASH more than the NAFLD condition [23]. In line with the results obtained by Kamikubo et al. [24], we found that BCP co-incubation can reduce the total lipid accumulation in a dose-dependent manner, with maximal activity at 5 µM. BCP was able to reduce both oleic and palmitic acid, here used to induce steatosis in HepG2 cells and also significantly reduced stearic and myristic acid, both saturated fatty acids associated with cellular damage [27,28]. Noticeably, BCP was able to induce a 37% increase in palmitoleic acid, a monounsaturated fatty acid that has been widely studied in in vivo models of obesity because of its anti-inflammatory properties [29]. We argue that the increase in palmitoleic acid might represent a protective detoxifying strategy converting palmitic acid into an unsaturated FFA. There is a general agreement that NAFLD progression occurs when mechanisms aimed at counteracting FFA-induced lipotoxicity are ineffective, leading to oxidative stress, ER stress, mitochondrial damage, immune-mediated cellular damage and apoptotic death [1]. Our data strongly suggest that BCP is able to reduce the amount of possibly toxic saturated fatty acids and increase selected monounsaturated fatty acids, thus representing a valuable agent in preventing cellular injuries associated with NASH. 

Besides emphasizing BCP ability to modify intracellular lipid composition, our data suggest that BCP effects involve the activation of CB2 as well as PPAR receptors.

PPARα and PPARγ are ligand-activated transcription factors with pleiotropic actions in several tissues. They are critical regulators not only of fatty acid metabolism, but also of glucose metabolism, inflammation and fibrosis [20]. The role of PPARα in the liver has been widely investigated both in physiological and steatotic conditions. The activation of PPARα induces the transcription of a range of genes involved in mitochondrial and peroxisomal FA oxidation, ketogenesis and lipid transport, thereby reducing hepatic lipid levels [20]. A recent study demonstrated that the deletion of hepatic *Pparα* in mice results in enhanced liver steatosis because of the impaired oxidation of FFA [30], underscoring the relevance and potential of hepatocyte PPARα as a drug target for NAFLD.

PPARγ is involved in FFA uptake and lipogenesis and has significant anti-inflammatory properties. Early results demonstrated that its activation is steatogenic in the liver [31] while recent works showed that PPARγ ligands (such as thiazolidinediones) ameliorate fat accumulation by decreasing saturated fatty acids in a zebrafish model of NAFLD [32]. This last effect possibly depends on the enhanced release of adiponectin by the adipose tissue and a concomitant increase in FFA oxidation in hepatocytes by AMPK activation as demonstrated in in vivo studies [33]. There are also in vitro studies demonstrating the role of PPARγ agonists in ameliorating lipid accumulation and inflammation associated with NASH. This is the case of GVS-12, a synthetic PPARγ agonist that can reduce triglycerides, inflammatory interleukins and other biomarkers associated with NASH in HepG2 cells [34]. An interesting synthetic ligand is saroglitazar, a dual PPARα/γ agonist with prevalent PPARα agonist activity. The efficacy of saroglitazar in counteracting NAFLD/NASH has been compared to that of fenofibrate, a PPARα agonist, and pioglitazone, a PPARγ agonist, showing that the combined action of saroglitazar improves lipid-mediated oxidative stress, inflammation and impaired mitochondrial biogenesis more effectively than single agonists, both in vitro and in vivo [35]. Many PPAR agonists, such as saroglitazar, are currently tested in clinical trials or have already been approved for the treatment of other metabolic diseases, such as pioglitazone used for the treatment of T2DM [3,20]. Ongoing clinical trials indicate that dual PPAR agonists can have ameliorating effects on NASH by acting on interrelated mechanisms. Thus, combining PPARα and PPARγ activation may be a successful strategy in the therapy of NAFLD [36].

According to our data, BCP effects are mediated both through PPARα and PPARγ. The activation of PPARα likely occurs through a direct mechanism; in support of this view, an interesting study demonstrated through a surface plasmon resonance (SPR)-BIA core system that BCP directly binds the PPARα ligand binding domain (LBD) even if it is a hydrophobic molecule and has a relatively small molecular weight compared with conventional PPAR ligands [13]. For PPARγ, there are no studies demonstrating a direct interaction with BCP; however, there is evidence of an indirect activation, possibly through CB2 receptors [22,37]. BCP dual activation of PPARα and PPARγ, observed in our in vitro experiments and previously shown in cocaine addiction studies performed in vivo [38], highlights the possibility that BCP might behave as a dual PPARα/γ agonist like saroglitazar. It should also be noted that GW9662, the PPARγ antagonist used in our experiments, has an IC50 value of 3.3 nM for PPARγ and 32 nM for PPARα [39]. Therefore, it cannot be excluded that GW9662 partially blocks PPARα, contributing to the reversion of the anti-steatotic effect of BCP observed in our experiments. In accordance with recently published results [40], we found that both PPARα and γ are downregulated during the pathological condition of steatosis. Intriguingly, co-treatment with BCP induced a marked upregulation of PPARα, bringing it back to even higher expression levels than in the untreated control. This latter result suggests that BCP might be able to enhance FA oxidation, therefore reducing hepatic steatosis.

The binding of BCP to the CB2 receptor has been well characterized [11], and the role of the endocannabinoid system (ECS) in the liver has been widely studied since it may be a therapeutic target for chronic liver disease [41], characterized by dysregulation of hepatic lipid metabolism and also perturbation of the hepatic endocannabinoid system [42]. Although CB2 expression in the liver is moderate, its role has been demonstrated in both physiological (regulating liver development in zebrafish embryos [43]) and pathological conditions. For example, recent studies showed that CB2, in contrast to CB1 [44], elicits anti-fibrogenic and anti-inflammatory effects [45]. However, the role of CB2 in the progression of NAFLD has been debated: on the one hand, studies on the activation of both CB1 and CB2 receptors have shown increased lipid accumulation [46] and potentiation of hepatic steatosis [47]; on the other hand, our results with a CB2 antagonist show that CB2 activation can counteract steatosis, in line with other works [24,48,49]. In agreement with in vivo data [50,51], we show that steatotic conditions upregulate CB2 expression in hepatocytes, and that concomitant exposure to BCP is able to revert the level of CB2 expression almost to control levels, thus facilitating return-to-normal conditions.

While PPARs are well known intracellular receptors, CB2 are seven-domain transmembrane receptors, whose localization is assumed to be on the plasma membrane. To verify this assumption, we performed immunolocalization studies. Unexpectedly, we found that CB2 receptors are located mainly intracellularly in HepG2 cells, often in a perinuclear position, most likely on the ER membrane or other intracellular organelles. Recent studies have suggested possible intracellular CB2 localization in specific cell types; for instance, Castaneda and colleagues [52] demonstrated that in peripheral blood B cells, CB2 receptor expression is regulated by different factors and these receptors are localized both on the cell membrane and on intracellular membranes. In line with this result, by kinetics studies, we showed for the first time that BCP can cross the hepatocyte plasma membrane and enter the cells with a maximum peak at 2 h, followed by a decrease, possibly due to BCP metabolism. Further studies are needed to determine the exact localization of intracellular CB2 receptors in hepatocytes and their involvement in the regulation of lipid metabolism.

## 4. Materials and Methods

### 4.1. Reagents

Chemicals used were: (E)-β-caryophyllene (BCP) purchased from Sigma-Aldrich (St. Louis, MO, USA), AdipoRed™ assay reagent from Lonza (Walkersville, MD, USA), NucBlue Live ReadyProbes Reagent from Invitrogen (Carlsbad, CA, USA), CellTiter-Glo^®^ Luminescent Cell Viability from Promega (Madison, WI, USA), anti-CB2 primary antibody from Cayman chemical (Ann Arbor, MI, USA), anti-Rabbit IgG AlexaFluor647 secondary antibody from Jackson Immunoresearch (Ely, UK), AM630, GW9662 and GW6741 antagonists from Cayman chemical and sodium oleate, sodium palmitate and bovine serum albumin from Sigma-Aldrich. Unless otherwise specified, all other chemicals were purchased from Sigma-Aldrich.

### 4.2. Cell Cultures

HepG2 human hepatoma cell line (European Collection of Authenticated Cell Cultures ECACC catalogue number 85011430) was purchased from Sigma-Aldrich. Cells were cultured in Minimum Essential Medium Eagle (MEM) supplemented with 10% fetal bovine serum, 2 mM L-glutamine, 50 IU/mL penicillin, 50 µg/mL streptomycin and 1% non-essential amino acids (NEAA). For every experiment, cells were grown at sub-confluence.

### 4.3. Cell Viability

The viability of HepG2 cells was evaluated at the end of the steatosis induction experiments and treated with different concentrations of BCP, by CellTiter-Glo^®^ Luminescent Cell Viability Assay, based on the quantitation of ATP, which signals the presence of metabolically active cells. Cells were washed in phosphate-buffered saline (PBS), then CellTiter-Glo^®^ reagent, diluted 1:1 in PBS, was added. Cells were incubated at room temperature in the dark for 10 min, then luminescence was detected and quantified with the FilterMax F5 Multi-Mode microplate reader (Molecular Devices, Sunnyvale, CA, USA). The values of luminescence are directly proportional to the number of viable cells. 

### 4.4. In Vitro Steatosis Induction and Lipid Quantification 

For steatosis induction experiments, 2 × 10^4^ cells/well were seeded in 96-well black clear bottom plates (Greiner Bio-One, Frickenhausen, Germany). Prior to the experiments, cells were starved overnight with serum-free MEM, then cells were incubated for 12 h or 24 h in serum-free MEM containing 0.25 mM, 0.5 mM or 1 mM free fatty acid mixture (FFAm). The mixture was prepared by coupling sodium palmitate (Na^+^-hexadecanoate) and sodium oleate (Na^+^-(Z)-octadec-9-enoate) (1:2 ratio) with 1% *w*/*v* FFA-free BSA in serum free MEM, at 38 °C in agitation overnight, to allow FFA coupling with BSA; the mixture was then filtered and used immediately in subsequent experiments or frozen at 20 °C. Based on the preliminary results we obtained, for subsequent experiments, HepG2 cells were treated for 24 h with 0.5 mM FFAm alone or in the presence of scalar dilutions of BCP (50 nM−50 μM); control cells were grown with serum-free MEM containing 1% *w*/*v* BSA. At the end of the experiments, cells were washed in PBS, then a dye mixture containing AdipoRed and NucBlue reagents (25 μL and 1 drop, respectively, for each mL of PBS) was added. AdipoRed assay reagent quantifies intracellular triglycerides, while the DNA content was estimated by NucBlue staining. After 40 min of incubation at room temperature in the dark, fluorescence was measured with Filtermax F5 microplate reader (Molecular Devices, San Jose, CA, USA); for AdipoRed, quantification excitation was performed at 485 nm and emission read at 535 nm, while for NucBlue, excitation was at 360 nm and emission read at 460 nm.

### 4.5. Antagonists Treatment

For antagonists experiments, 2 × 10^4^ cells/well were seeded in 96-well black clear bottom plates, starved and treated as above mentioned with FFA mixture, 5 µM BCP and the following antagonists: 5 µM AM630 (CB2 receptor antagonist), 100 nM GW6471 (PPARα receptor antagonist) or 10 µM GW9662 (PPARγ receptor antagonist). 

### 4.6. Lipid Extraction, Identification and Quantification by Gas Chromatography

For lipid extraction and quantification experiments, 2 × 10^6^ cells were grown on T-25 flasks. After cell starvation, HepG2 cells were treated with FFAm alone or FFAm and 5 µM BCP for 24 h. One ml of culture medium was then taken for gas chromatography–mass spectrometry (GC-MS) analysis of free fatty acids. The intracellular fatty acid content was also analyzed; cells were washed with PBS, detached with trypsin and centrifuged at 800 g, 5 min. Lipids were extracted from cell pellets using cyclohexane (1:10, *w*/*v* ratio) and then esterified with boron tri-fluoride (10% *w*/*v* in methanol). Fifty μg heptadecanoic acid (C17:0) was added as the internal standard. Fatty acid methyl esters (FAME) identification was performed by gas chromatography coupled with mass spectrometry (GC-MS) (5975T, Agilent Technologies, Santa Clara, CA, USA). FAME quantitative analyses were performed through GC coupled with a flame ionization detector (GC-FID) (GC-2010 Plus, SHIMADZU, Kyoto, Japan). The GC carrier gas was helium with a constant flux of 1 mL min^−1^, and separation was obtained with a non-polar capillary column ZB5-MS (30 m length, 250 μm diameter and stationary phase thickness of 0.25 μm, 5% phenyl-arylene and 95% poly-dimethyl siloxane stationary phase) (Phenomenex, Torrance, CA, USA). GC-FID FAME separation was performed in the same conditions, by using a similar column. Mass spectrometer parameters were: ionization energy of the ion source set to 70 eV and the acquisition mode set to 50–350 *m/z*. Separated molecules were identified through the comparison of mass fragmentation spectra with reference spectra of the software NIST v2.0 and libraries NIST 98, by comparison of Kovats indexes and the internal standard injection (C17,C20:4, C20:5—Sigma Aldrich, St Louis, MO, USA). The results are expressed as mg g^−1^ fresh weight (f.wt).

### 4.7. Intracellular Quantification of BCP in Time-Course Experiments

For BCP time-course uptake experiments, 1 × 10^6^ cells were grown on 6-well plates and treated with 5 μM BCP. Starting from time zero and after 30 min, 1 h, 1.5 h, 2 h, 3 h, 4 h, 5 h, 6 h, 10 h and 24 h cells were washed in PBS, detached with trypsin, centrifuged and the pellets were transferred in glass vials where 1 mL of hexane was added. The identification of BCP amounts in cells at different times was performed with the Single Ion Monitoring (SIM) method by GC-MS. The following chromatographic conditions were used: column ZB5-MS (30 m length, 250 μm diameter and stationary phase thickness of 0.25 μm, 5% phenyl-arylene and 95% of poly-dimethyl siloxane stationary phase); splitless mode, oven program: 40° for 1 min, then a 5 °C min^−1^ ramp to 200 °C, a 10 °C min^−1^ ramp to 220 °C, and a 30 °C min^−1^ ramp to 260 °C, final temperature held for 3.6 min. Mass spectra were acquired within the 29–350 m/z interval operating the spectrometer at 70 eV and at scan speed mode. The identification of BCP was performed on the basis of both matches of the peak spectra with a library spectral database, and comparison with pure standards.

### 4.8. Immunofluorescence 

For immunofluorescence experiments, 6 × 10^4^ cell/cm^2^ were seeded on glass coverslips and incubated for at least 3 h to allow adhesion. Then, cells were fixed for 30 min in 4% paraformaldehyde dissolved in 0.1 M phosphate buffer, pH 7.3. After three washes with PBS, cells were incubated for 15 min with PBS containing 0.01% Triton-X100 and then for 45 min with PBS containing 1% BSA and 10% normal donkey serum. Triton-X100, normal donkey serum and BSA concentrations were selected after several trials to avoid autofluorescence of HepG2. Cells were then incubated overnight at 4 °C with anti-CB2 (1:100 in PBS) primary polyclonal antibody. Coverslips were washed twice with PBS and incubated for 1 h at room temperature with the secondary antibody, anti-rabbit IgG Alexa Fluor 647 (1:600 in PBS containing 1% normal donkey serum). After two washes in PBS, cells were incubated for 20 min in DAPI (1:200 in PBS), washed again twice in PBS and then coverslips were mounted on standard slides with DABCO. Pictures of HepG2 cells immunolabeled for CB2 were taken with a TCS SP5 confocal microscope (Leica Microsystems, Wetzlar, Germany). Confocal image z-stacks were captured throughout the thickness of the cells and were performed with 0.7 µm optical step size using an objective 63X/1.4 NA oil immersion lens with a resolution of 8-bit, 1024/1024 pixels and 100-Hertz scan-speed (without additional zoom: 1 voxel, xyz = 240 nm × 240 nm × 692 nm; with 2.5× additional zoom: 1 voxel, xyz = 96 nm × 96 nm × 692 nm). Images are shown as maximum intensity projection or single plane with reslice.

### 4.9. RNA Extraction and qRT-PCR

For qRT-PCR experiments, 1 × 10^6^ cells were grown on 6-well plates and treated to induce steatosis in the presence/absence of 5 µM BCP (see above). Total RNA extraction was performed using TRIZOL^®^ Reagent (Invitrogen) following manufacturer’s instructions. Chloroform was added and, after 5 min of centrifugation at 14,000 rpm, RNA was precipitated in isopropanol for 3 h at −20 °C. Samples were then centrifuged at 14,000 rpm for 15 min, the RNA was washed with 70% ethanol and centrifuged at 14,000 rpm for 5 min. The RNA pellet was briefly air-dried and resuspended in 30 µL sterile water. Samples were quantified using NanoDrop 8000 Spectrophotometer (Thermo Fisher Scientific, Waltham, MA, USA). qReal-time PCR was performed using SensiFast SYBR No-ROX One-Step Real-Time qPCR kit (Bioline, London, UK) in the thermal cycler Rotor Gene Q (Qiagen, Hilden, Germany). qRT-PCR conditions were: retrotranscription (55 °C, 15 min), initial denaturation (95 °C, 2 min), 50 cycles of denaturation (94 °C, 15 s), annealing (55 °C, 10 s), extension (68 °C, 24 s) and final melting (ramp from 56 °C to 99 °C). Each RNA sample was analyzed in three technical replicates containing 50 ng of total RNA. Relative quantification of mRNA abundance in each sample was performed using a standard curve, built with several dilutions of the samples. The widely used housekeeping gene β-actin was used as an internal control to normalize target gene expression. The reliability of the housekeeping gene was confirmed by its consistency of expression across treatments. qRT-PCR starting from constant amounts (50 ng) of different RNA samples, accurately quantified by NanoDrop Spectrophotometer analysis, resulted in fact in comparable levels of amplification (Ct, cycle threshold values). Specific primers were designed with PrimerBlast software on the basis of human sequences (Table 1). The qPCR primer efficiencies were first assessed by the amplification of serial dilutions of RNA pools (three replicates for each dilution); the efficiency values were directly calculated by Rotor Gene Q software and were as follows: β-actin 98%, *CNR2* 103%, *PPARα* 95%, *PPARγ* 105%. 

Primers sequences and amplicon size are reported in Table 1

### 4.10. Statistical Analysis

All experimental data are presented as means ± standard error of the mean (SEM) from at least three technical replicates of 3–5 independent biological experiments (the exact number of independent experiments is indicated in the figure legends). Statistical analysis was performed using the SPSS package version 28. Statistically significant differences between treatment and control groups were assessed by a one-way analysis of variance (ANOVA) followed by Bonferroni’s multiple-comparison post hoc test. Differences were considered statistically significant at *p <* 0.05.

## 5. Conclusions

Taken together, our results suggest that BCP is a promising molecule for the treatment of NAFLD. This conclusion is based on several key aspects of this molecule. BCP may act on multiple targets, many of which are included in NAFLD and metabolic syndrome, since it is able to reduce lipid accumulation in hepatocytes but also in adipocytes [53], may improve muscle insulin resistance [12] and systemic inflammation, therefore resulting in a greater overall improvement compared with compounds with a more liver-restricted mode of action. It should also be considered that BCP is an approved dietary additive with a good safety profile and a safe phytocannabinoid, since it binds specifically to CB2 receptors, thus avoiding the psychotropic effects mediated by CB1 receptors [54]. There are, however, issues related to BCP bioavailability; in fact some studies have already focused on alternative formulations and vectorization techniques to allow better absorption, overcoming the limitations of BCP and making the most of all the properties of this phytocannabinoid [55,56]. 

## Figures and Tables

**Table 1:** Specific primers used in qRT-PCR.

Gene	Forward Sequence	Reverse Sequence	Amplicon Size	GenBank Accession Number
β-actin	5′ CCAACCGCGAGAAGATGA 3′	5′ CCAGAGGCGTACAGGGATAG 3′	97 bp	NM_001101.5
*CNR2*	5′ TGGCATAGAAGACGGAGCTG 3′	5′ CCCGGAGAGCCCCAAATG 3′	177 bp	NM_001841.3
*PPARα*	5′ ACACCGAGGACTCTTGCGA 3′	5′ GGAAAGGGCAAGTCCCGATG 3′	207 bp	NM_001393944.1
*PPAR* *γ*	5′ TACTGTCGGTTTCAGAAATGCC 3′	5′ GTCAGCGGACTCTGGATTCAG 3′	141 bp	NM_138712.5

## Data Availability

Data are available upon request.

## References

[1] Powell E.E., Wong V.W.-S., Rinella M. (2021). Non-Alcoholic Fatty Liver Disease. Lancet.

[2] Friedman S.L., Neuschwander-Tetri B.A., Rinella M., Sanyal A.J. (2018). Mechanisms of NAFLD Development and Therapeutic Strategies. Nat. Med..

[3] Mantovani A., Dalbeni A. (2021). Treatments for NAFLD: State of Art. Int. J. Mol. Sci..

[4] Geethangili M., Lin C.-W., Mersmann H.J., Ding S.-T. (2021). Methyl Brevifolincarboxylate Attenuates Free Fatty Acid-Induced Lipid Metabolism and Inflammation in Hepatocytes through AMPK/NF-ΚB Signaling Pathway. Int. J. Mol. Sci..

[5] Zhu X., Bian H., Wang L., Sun X., Xu X., Yan H., Xia M., Chang X., Lu Y., Li Y. (2019). Berberine Attenuates Nonalcoholic Hepatic Steatosis through the AMPK-SREBP-1c-SCD1 Pathway. Free Radic. Biol. Med..

[6] Maffei M.E. (2020). Plant Natural Sources of the Endocannabinoid (E)-β-Caryophyllene: A Systematic Quantitative Analysis of Published Literature. Int. J. Mol. Sci..

[7] Scandiffio R., Geddo F., Cottone E., Querio G., Antoniotti S., Gallo M.P., Maffei M.E., Bovolin P. (2020). Protective Effects of (E)-β-Caryophyllene (BCP) in Chronic Inflammation. Nutrients.

[8] Mannino F., Pallio G., Corsaro R., Minutoli L., Altavilla D., Vermiglio G., Allegra A., Eid A.H., Bitto A., Squadrito F. (2021). Beta-Caryophyllene Exhibits Anti-Proliferative Effects through Apoptosis Induction and Cell Cycle Modulation in Multiple Myeloma Cells. Cancers.

[9] Yovas A., Manjusha W.A., Ponnian S.M.P. (2022). β-Caryophyllene Modulates B-Cell Lymphoma Gene-2 Family Genes and Inhibits the Intrinsic Pathway of Apoptosis in Isoproterenol-Induced Myocardial Infarcted Rats; A Molecular Mechanism. Eur. J. Pharmacol..

[10] Picciolo G., Pallio G., Altavilla D., Vaccaro M., Oteri G., Irrera N., Squadrito F. (2020). β-Caryophyllene Reduces the Inflammatory Phenotype of Periodontal Cells by Targeting CB2 Receptors. Biomedicines.

[11] Gertsch J., Leonti M., Raduner S., Racz I., Chen J.Z., Xie X.Q., Altmann K.H., Karsak M., Zimmer A. (2008). Beta-Caryophyllene Is a Dietary Cannabinoid. Proc. Natl. Acad. Sci. USA.

[12] Geddo F., Antoniotti S., Querio G., Salaroglio I.C., Costamagna C., Riganti C., Gallo M.P. (2021). Plant-Derived Trans-β-Caryophyllene Boosts Glucose Metabolism and ATP Synthesis in Skeletal Muscle Cells through Cannabinoid Type 2 Receptor Stimulation. Nutrients.

[13] Wu C., Jia Y., Lee J.H., Jun H.J., Lee H.S., Hwang K.Y., Lee S.J. (2014). Trans-Caryophyllene Is a Natural Agonistic Ligand for Peroxisome Proliferator-Activated Receptor-α. Bioorganic Med. Chem. Lett..

[14] Irrera N., D’ascola A., Pallio G., Bitto A., Mazzon E., Mannino F., Squadrito V., Arcoraci V., Minutoli L., Campo G.M. (2019). β-Caryophyllene Mitigates Collagen Antibody Induced Arthritis (CAIA) in Mice Through a Cross-Talk between CB2 and PPAR-γ Receptors. Biomolecules.

[15] Charytoniuk T., Zywno H., Berk K., Bzdega W., Kolakowski A., Chabowski A., Konstantynowicz-Nowicka K. (2022). The Endocannabinoid System and Physical Activity—A Robust Duo in the Novel Therapeutic Approach against Metabolic Disorders. Int. J. Mol. Sci..

[16] Lowe H., Toyang N., Steele B., Bryant J., Ngwa W. (2021). The Endocannabinoid System: A Potential Target for the Treatment of Various Diseases. Int. J. Mol. Sci..

[17] Machado K.d.C., Islam M.T., Ali E.S., Rouf R., Uddin S.J., Dev S., Shilpi J.A., Shill M.C., Reza H.M., Das A.K. (2018). A Systematic Review on the Neuroprotective Perspectives of Beta-Caryophyllene.

[18] Mlost J., Wąsik A., Starowicz K. (2019). Role of Endocannabinoid System in Dopamine Signalling within the Reward Circuits Affected by Chronic Pain. Pharmacol. Res..

[19] Francomano F., Caruso A., Barbarossa A., Fazio A., La Torre C., Ceramella J., Mallamaci R., Saturnino C., Iacopetta D., Sinicropi M.S. (2019). β-Caryophyllene: A Sesquiterpene with Countless Biological Properties. Appl. Sci..

[20] Berthier A., Johanns M., Zummo F.P., Lefebvre P., Staels B. (2021). PPARs in Liver Physiology. Biochim. Biophys. Acta Mol. Basis Dis..

[21] Wu L., Guo C., Wu J. (2020). Therapeutic Potential of PPARγ Natural Agonists in Liver Diseases. J. Cell. Mol. Med..

[22] Youssef D.A., El-Fayoumi H.M., Mahmoud M.F. (2019). Beta-Caryophyllene Protects against Diet-Induced Dyslipidemia and Vascular Inflammation in Rats: Involvement of CB2 and PPAR-γ Receptors. Chem. -Biol. Interact..

[23] Gómez-Lechón M.J., Donato M.T., Martínez-Romero A., Jiménez N., Castell J.V., O’Connor J.-E. (2007). A Human Hepatocellular in Vitro Model to Investigate Steatosis. Chem.-Biol. Interact..

[24] Kamikubo R., Kai K., Tsuji-Naito K., Akagawa M. (2016). β-Caryophyllene Attenuates Palmitate-Induced Lipid Accumulation through AMPK Signaling by Activating CB2 Receptor in Human HepG2 Hepatocytes. Mol. Nutr. Food Res..

[25] Ma Z.-G., Yuan Y.-P., Zhang X., Xu S.-C., Wang S.-S., Tang Q.-Z. (2017). Piperine Attenuates Pathological Cardiac Fibrosis Via PPAR-γ/AKT Pathways. EBioMedicine.

[26] Atanasov A.G., Zotchev S.B., Dirsch V.M., Supuran C.T. (2021). Natural Products in Drug Discovery: Advances and Opportunities. Nat. Rev. Drug Discov..

[27] Saraswathi V., Kumar N., Ai W., Gopal T., Bhatt S., Harris E.N., Talmon G.A., Desouza C.V. (2022). Myristic Acid Supplementation Aggravates High Fat Diet-Induced Adipose Inflammation and Systemic Insulin Resistance in Mice. Biomolecules.

[28] Lu H., Guo R., Zhang Y., Su S., Zhao Q., Yu Y., Shi H., Sun H., Zhang Y., Li S. (2021). Inhibition of LncRNA TCONS_00077866 Ameliorates the High Stearic Acid Diet-Induced Mouse Pancreatic β-Cell Inflammatory Response by Increasing MiR-297b-5p to Downregulate SAA3 Expression. Diabetes.

[29] Simão J.J., Cruz M.M., Abdala F.M., Bolsoni-Lopes A., Armelin-Correa L., Alonso-Vale M.I.C. (2022). Palmitoleic Acid Acts on Adipose-Derived Stromal Cells and Promotes Anti-Hypertrophic and Anti-Inflammatory Effects in Obese Mice. Pharmaceuticals.

[30] Montagner A., Polizzi A., Fouché E., Ducheix S., Lippi Y., Lasserre F., Barquissau V., Régnier M., Lukowicz C., Benhamed F. (2016). Liver PPARα Is Crucial for Whole-Body Fatty Acid Homeostasis and Is Protective against NAFLD. Gut.

[31] Yu S., Matsusue K., Kashireddy P., Cao W.-Q., Yeldandi V., Yeldandi A.V., Rao M.S., Gonzalez F.J., Reddy J.K. (2003). Adipocyte-Specific Gene Expression and Adipogenic Steatosis in the Mouse Liver Due to Peroxisome Proliferator-Activated Receptor Gamma1 (PPARgamma1) Overexpression. J. Biol. Chem..

[32] Singh M.K., Yadav R., Bhaskar A.K., Sengupta S., Sachidanandan C. (2023). A Diet-Independent Zebrafish Model for NAFLD Recapitulates Patient Lipid Profiles and Offers a System for Small Molecule Screening. Biochim. et Biophys. Acta (BBA)—Mol. Cell. Biol. Lipids.

[33] Wang Y., Nakajima T., Gonzalez F.J., Tanaka N. (2020). PPARs as Metabolic Regulators in the Liver: Lessons from Liver-Specific PPAR-Null Mice. Int. J. Mol. Sci..

[34] Wang Y., Zhang X., Yuan B., Lu X., Zheng D., Zhang K., Zhong M., Xu X., Duan X. (2019). GVS-12 Attenuates Non-Alcoholic Steatohepatitis by Suppressing Inflammatory Responses via PPARγ/STAT3 Signaling Pathways. RSC Adv..

[35] Jain M.R., Giri S.R., Bhoi B., Trivedi C., Rath A., Rathod R., Ranvir R., Kadam S., Patel H., Swain P. (2018). Dual PPARα/γ Agonist Saroglitazar Improves Liver Histopathology and Biochemistry in Experimental NASH Models. Liver Int..

[36] Francque S., Szabo G., Abdelmalek M.F., Byrne C.D., Cusi K., Dufour J.-F., Roden M., Sacks F., Tacke F. (2021). Nonalcoholic Steatohepatitis: The Role of Peroxisome Proliferator-Activated Receptors. Nat. Rev. Gastroenterol. Hepatol..

[37] Cheng Y., Dong Z., Liu S. (2014). β-Caryophyllene Ameliorates the Alzheimer-like Phenotype in APP/PS1 Mice through CB2 Receptor Activation and the PPARγ Pathway. Pharmacology.

[38] Galaj E., Bi G.-H., Moore A., Chen K., He Y., Gardner E., Xi Z.-X. (2021). Beta-Caryophyllene Inhibits Cocaine Addiction-Related Behavior by Activation of PPARα and PPARγ: Repurposing a FDA-Approved Food Additive for Cocaine Use Disorder. Neuropsychopharmacology.

[39] Indrayanto G., Putra G.S., Suhud F., Al-Majed A.A. (2021). Chapter Six—Validation of in-Vitro Bioassay Methods: Application in Herbal Drug Research. Profiles of Drug Substances, Excipients and Related Methodology.

[40] Songtrai S., Pratchayasakul W., Arunsak B., Chunchai T., Kongkaew A., Chattipakorn N., Chattipakorn S.C., Kaewsuwan S. (2022). Cyclosorus Terminans Extract Ameliorates Insulin Resistance and Non-Alcoholic Fatty Liver Disease (NAFLD) in High-Fat Diet (HFD)-Induced Obese Rats. Nutrients.

[41] Basu P.P., Aloysius M.M., Shah N.J., Brown R.S. (2014). Review Article: The Endocannabinoid System in Liver Disease, a Potential Therapeutic Target. Aliment. Pharmacol. Ther..

[42] Mboumba Bouassa R.-S., Sebastiani G., Di Marzo V., Jenabian M.-A., Costiniuk C.T. (2022). Cannabinoids and Chronic Liver Diseases. Int. J. Mol. Sci..

[43] Liu L.Y., Alexa K., Cortes M., Schatzman-Bone S., Kim A.J., Mukhopadhyay B., Cinar R., Kunos G., North T.E., Goessling W. (2016). Cannabinoid Receptor Signaling Regulates Liver Development and Metabolism. Development.

[44] Jorgačević B., Vučević D., Samardžić J., Mladenović D., Vesković M., Vukićević D., Ješić R., Radosavljević T. (2021). The Effect of CB1 Antagonism on Hepatic Oxidative/Nitrosative Stress and Inflammation in Nonalcoholic Fatty Liver Disease. Curr. Med. Chem..

[45] Julien B., Grenard P., Teixeira-Clerc F., Van Nhieu J.T., Li L., Karsak M., Zimmer A., Mallat A., Lotersztajn S. (2005). Antifibrogenic Role of the Cannabinoid Receptor CB2 in the Liver. Gastroenterology.

[46] De Gottardi A., Spahr L., Ravier-Dall’Antonia F., Hadengue A. (2010). Cannabinoid Receptor 1 and 2 Agonists Increase Lipid Accumulation in Hepatocytes. Liver Int..

[47] Deveaux V., Cadoudal T., Ichigotani Y., Teixeira-Clerc F., Louvet A., Manin S., Nhieu J.T.-V., Belot M.P., Zimmer A., Even P. (2009). Cannabinoid CB2 Receptor Potentiates Obesity-Associated Inflammation, Insulin Resistance and Hepatic Steatosis. PLoS ONE.

[48] Bazwinsky-Wutschke I., Zipprich A., Dehghani F. (2019). Endocannabinoid System in Hepatic Glucose Metabolism, Fatty Liver Disease, and Cirrhosis. Int. J. Mol. Sci..

[49] Baldassarre M., Giannone F.A., Napoli L., Tovoli A., Ricci C.S., Tufoni M., Caraceni P. (2013). The Endocannabinoid System in Advanced Liver Cirrhosis: Pathophysiological Implication and Future Perspectives. Liver Int..

[50] Rivera P., Vargas A., Pastor A., Boronat A., López-Gambero A.J., Sánchez-Marín L., Medina-Vera D., Serrano A., Pavón F.J., de la Torre R. (2020). Differential Hepatoprotective Role of the Cannabinoid CB1 and CB2 Receptors in Paracetamol-Induced Liver Injury. Br. J. Pharmacol..

[51] Mendez-Sanchez N., Zamora-Valdes D., Pichardo-Bahena R., Barredo-Prieto B., Ponciano-Rodriguez G., Bermejo-Martínez L., Chavez-Tapia N.C., Baptista-González H.A., Uribe M. (2007). Endocannabinoid Receptor CB2 in Nonalcoholic Fatty Liver Disease. Liver Int..

[52] Castaneda J.T., Harui A., Roth M.D. (2017). Regulation of Cell Surface CB2 Receptor during Human B Cell Activation and Differentiation. J. Neuroimmune Pharm..

[53] Geddo F., Scandiffio R., Antoniotti S., Cottone E., Querio G., Maffei M.E., Bovolin P., Gallo M.P. (2019). Pipenig^®^-FL, a Fluid Extract of Black Pepper (*Piper Nigrum* L.) with a High Standardized Content of Trans-β-Caryophyllene, Reduces Lipid Accumulation in 3T3-L1 Preadipocytes and Improves Glucose Uptake in C2C12 Myotubes. Nutrients.

[54] Hempel B., Xi Z.-X. (2022). Receptor Mechanisms Underlying the CNS Effects of Cannabinoids: CB1 Receptor and Beyond. Adv. Pharm..

[55] Santos P.S., Oliveira T.C., Júnior L.M.R., Figueiras A., Nunes L.C.C. (2018). β-Caryophyllene Delivery Systems: Enhancing the Oral Pharmacokinetic and Stability. Curr. Pharm. Des..

[56] Mödinger Y., Knaub K., Dharsono T., Wacker R., Meyrat R., Land M.H., Petraglia A.L., Schön C. (2022). Enhanced Oral Bioavailability of β-Caryophyllene in Healthy Subjects Using the VESIsorb^®^ Formulation Technology, a Novel Self-Emulsifying Drug Delivery System (SEDDS). Molecules.

